# A cadaveric study of the location and morphology of the central patellar ridge for bone-patellar tendon-bone graft

**DOI:** 10.1186/s13018-021-02244-5

**Published:** 2021-01-28

**Authors:** Thanathep Tanpowpong, Thun Itthipanichpong, Thanasil Huanmanop, Nonn Jaruthien, Nattapat Tangchitcharoen

**Affiliations:** 1 Department of Orthopaedics, Chulalongkorn University, King Chulalongkorn Memorial Hospital, Thai Red Cross society, Bangkok, Thailand; 2 Department of Anatomy, Chulalongkorn University, Bangkok, Thailand

**Keywords:** Patellar fracture, ACL reconstruction, Bone-patellar tendon-bone, Wiberg’s classification, Patellar morphology

## Abstract

**Introduction:**

The central ridge of the patella is the thickest area of patella and varies among patients. This cadaveric study identified the location and thickness of the bone at the central patella ridge for bone-patellar tendon-bone (BPTB) harvesting.

**Materials and methods:**

Fifty cadaveric knees were assessed. First, the morphology, length, width, and location of the central patellar ridge were recorded. Then, we transversely cut the patella 25 mm from the lower pole and measured the thickness of the anterior cortex, cancellous bone, and cartilage from both the mid-patella and the central ridge location. Finally, the depth of the remaining cancellous bone at the mid-patella was compared to the bone at the central ridge.

**Results:**

The location of the central-patellar ridge deviated medially from the mid-patella in 46 samples with an average distance of 4.36 ± 1 mm. Only 4 samples deviated laterally. The mean patella length was 41.19 ± 4.73 mm, and the width was 42.8 ± 5.25 mm. After a transverse cut, the remaining cancellous bone was significantly thicker at the central ridge compared to the bone at the mid-patella.

**Conclusions:**

Most of the central patellar ridge deviated medially, approximately 4 mm from the mid-patella. Harvesting the graft from the central ridge would have more remaining bone compared to the mid-patella.

## Introduction

Knee ligament injuries are common sports injury, especially in young athletes. Ligament reconstruction is often needed with the goal of returning the knee to its pre-injury status. Bone-patellar tendon-bone (BPTB) is one of the grafts commonly used for ligament reconstruction. Complications from BPTB harvesting are anterior knee pain, non-union patella [1], and patellar fracture [2–7]. Although patellar fracture is rare, it is a devastating complication and can lead to poor outcome in a young athlete [6]. There were many reports of patellar fracture following middle one-third harvesting [2–4, 6, 7] and medial one-third patellar harvesting [5]. It is difficult to identify this complication because many different surgical techniques were used. BPTB harvesting technique usually involves 20–30 mm of the bone from the lower pole of the patella with less than 10 mm of depth in triangular shape [8].

**Fig. 1:**
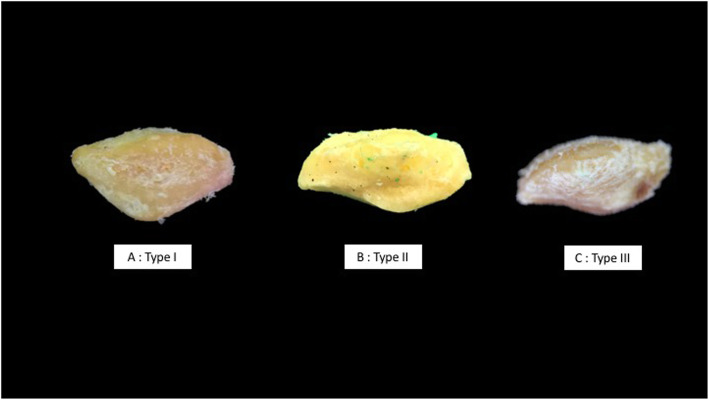
Anatomy picture of the patella according to Wiberg’s classification. **a** Type I, the facets are concave, symmetrical, and of equal size. **b** Type II, the medial facet is smaller than the lateral facet. **c** Type III, the convex medial facet is markedly smaller than the concave lateral facet, and the angle between the medial and lateral facets is nearly 90°

We believe that understanding the patellar morphology has an important role and can help us avoid such complication. Originally, Wiberg [9] classified the patellar morphology into 3 types according to the axial radiographic view. For type I, the facets are concave, symmetrical, and of equal size. For type II, the medial facet is smaller than the lateral facet (medial facet is flat or slightly convex and lateral facet is concave). As for type III, the convex medial facet is markedly smaller than the concave lateral facet, and the angle between the medial and lateral facets is nearly 90° (Fig. 1). Additional classification (Wiberg IV) defines that there were no medial facet and central ridge [10]. We believe that the thickest part of the patella should be at the central ridge area. The traditional technique for BPTB harvesting from mid-patella may cause weakness of the harvesting site in groups of patients whose central patellar ridge is not in the midline position. This might be one of the causes for patella fracture after BPTB harvesting. We conducted a study to identify the morphology of the patella and the location of the central patellar ridge. We believe that harvesting BPTB at the central ridge should leave us with more bone compared to the bones obtained from the mid-patella.

## Materials and methods

### Data collection

An institutional review board approval was obtained before initiation of the study. We examined 50 cadaveric patellae from 50 cadavers at our hospital. We excluded cadavers with damaged patella, bipartite patellae, or patella with odd facet because it cannot be measured properly. The evaluation and measurement of all specimens were done by 3 separate investigators. If one of the investigators suggested that the bone was not in optimum condition, we discarded that specimen.

First, surgical dissection was done to remove the patella from the cadaver. All soft tissue and periosteum were removed from the patella. Measurement was done using a digital caliper (Becker model EC10, Mumbai, India).

**Fig. 2:**
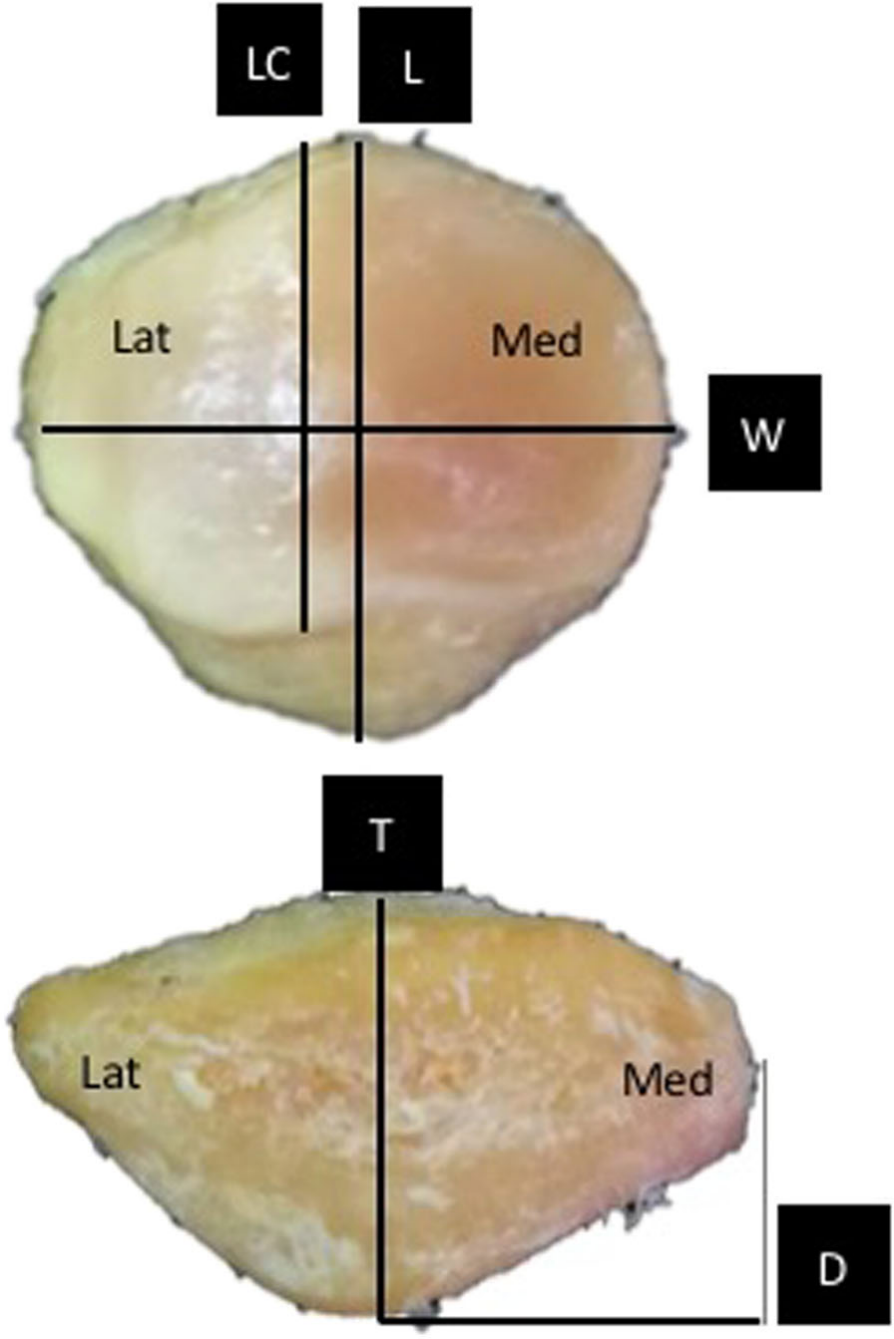
Methods of measuring patellar morphology. Lat, lateral; Med, medial; L, length of patella; LC, length of cartilage; W, width of patella; T, thickness of patella; D, distance from medial border to central-ridge

**Fig. 3:**
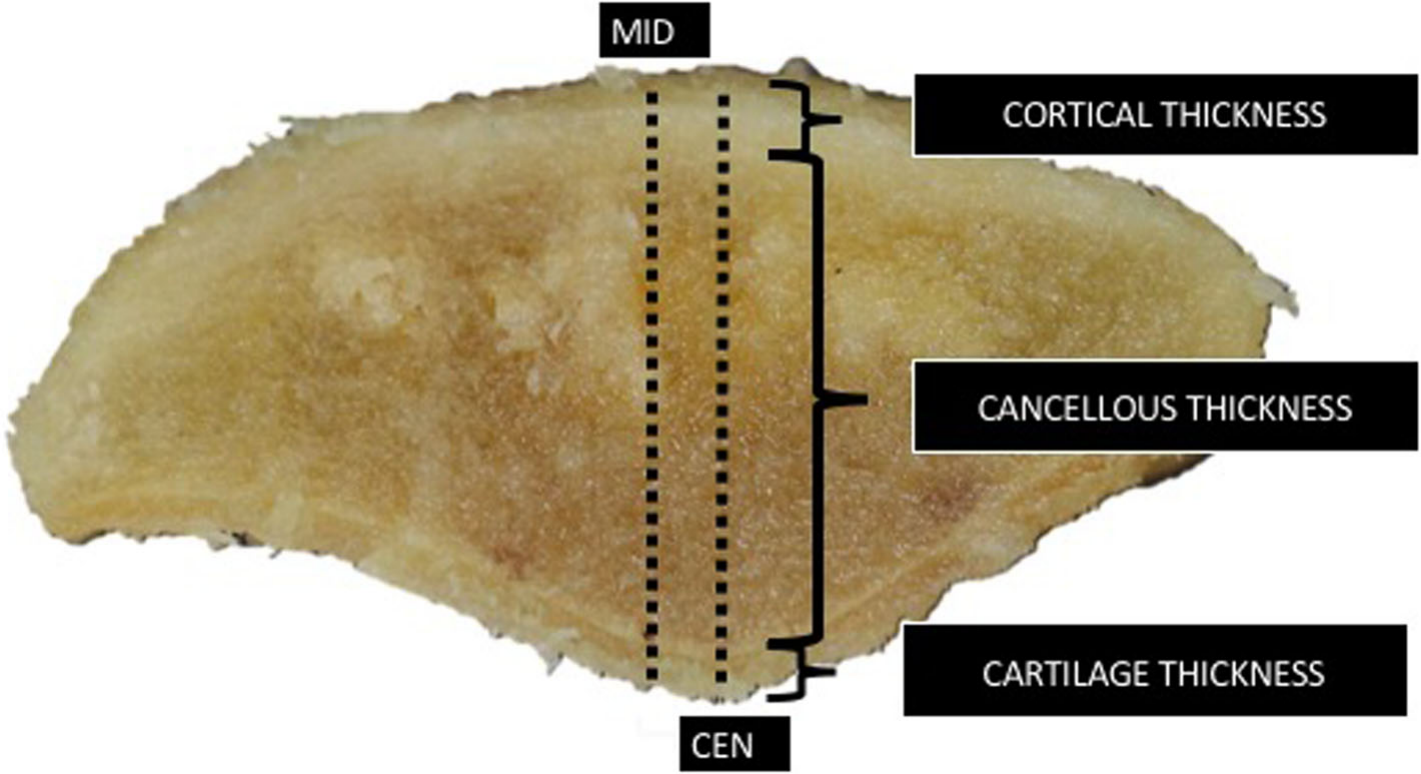
After cutting 25 mm above the lower pole of the patella. Parameters were measured. MID, mid-patella; CEN, central patellar ridge

Measurements of the length, width, thickness, distance from medial border of patella to the central ridge, and length of patellar cartilage were obtained. Average distance from the mid-patella to the central ridge was calculated (Fig. 2). After the measurement was done, we transversely cut the patella 25 mm from the lower pole which is the length usually used for BPTB harvesting, and measured the thickness of the anterior cortex, cancellous, cartilage at mid-patella, and central ridge. Using the standard harvesting technique, approximately 10-mm thickness of bone was removed, and we compared the depth of the remaining cancellous bone after harvesting the BPTB from the mid-patella with that from the central ridge (Fig. 3).

### Statistical analysis

The reliability was calculated using the intraclass correlation coefficient (ICC). Descriptive statistic was presented as mean and standard deviation (SD). Statistical analyses were calculated with SPSS version 22. Paired *t* test was used for mean comparison of the normal distributions of the continuous variables. A *P* value of less than 0.05 was considered to be statistically significant.

## Results

The ICCs for intra- and inter-rater agreements were > 0.90 for all measurements. The ICCs value of > 0.80 was defined to be an excellent agreement.

**Fig. 4:**
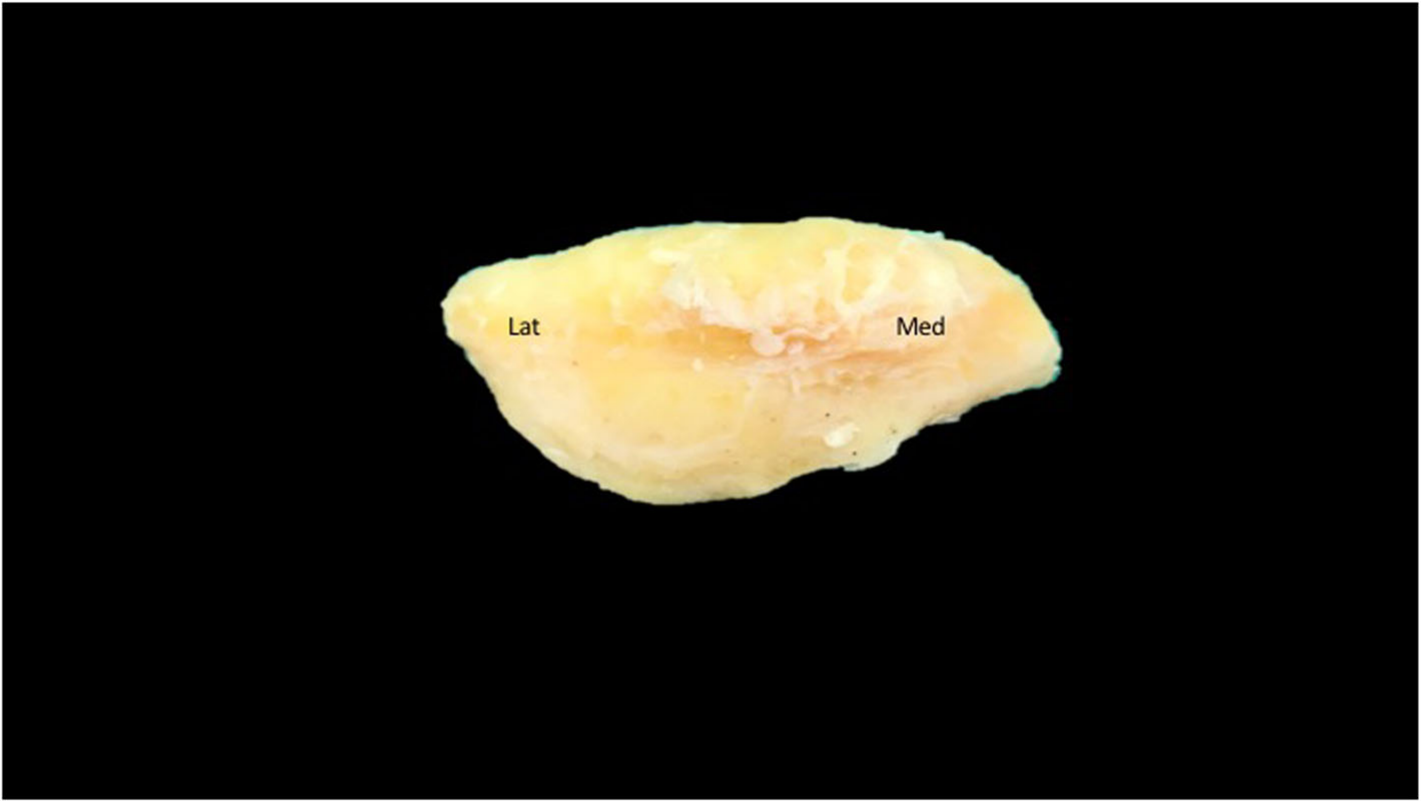
Picture of the right patella which shows that the medial facet was larger than the lateral facet

Fourteen percent (*n* = 7), 68% (*n* = 34), and 12% (*n* = 6) were Wiberg types I, II, and III, respectively. We found 3 unclassified samples (6%) which had a larger medial facet compared to the lateral facet (Table 1 and Fig. 4). The mean patella length, width, and distance from the medial border to the central ridge were 41.19 ± 4.73 mm, 42.8 ± 5.25 mm, and 17.78 ± 2.96 mm, respectively (Table 2).

**Table 1:** Patellar Wiberg’s classification

Wiberg’s classification	Percent (%)
Type I (*n* = 18)	36
Type II (*n* = 23)	46
Type III (*n* = 6)	12
Unclassified (type IV) (*n* = 3)	6

**Table 2:** Overall patellar morphology

	Mean (mm)	SD	Max	Min
Longitudinal length (L)	41.19	4.73	53.18	33.13
Longitudinal length of cartilage (LC)	32.75	3.58	45.78	26.26
Width of patella (W)	42.8	5.25	53.42	21.7
Patellar thickness (T)	21.63	2.08	26.78	17.7
Distance from medial border to central ridge (D)	17.78	2.96	26.44	12.07

The central ridge located at the medial, middle, and lateral one-third of the patella was 6% (*n* = 3), 88% (*n* = 44), and 6% (*n* = 3), respectively. The mean deviated distance from the mid-patella to medially deviated central patella ridge was 4.36 ± 1 (*n* = 46) and from mid-patella to laterally deviated patella ridge (*n* = 4) was 4.9 ± 2.86 mm.

After a transverse cut, the average thickness of the anterior cortex, cancellous bone, and cartilage at the mid patella location was 4.04 ± 1.01 mm, 14.26 ± 2.12 mm, and 1.08 ± 0.46 mm, respectively. The average thickness at the central patellar ridge level was 4.77 ± 0.98 mm, 15.54 ± 2.09 mm, and 1.55 ± 0.65 mm, respectively (Table 3). The average thickness of the anterior cortex, cancellous, and cartilage at the central ridge level was significantly thicker than at the mid-patella level (*p* < 0.010) (Table 4). The remaining cancellous thickness after harvesting 10-mm depth of BPTB was 8.3 ± 2.04 mm in the mid-patella and 10.31 ± 2.23 mm in the central ridge region (*p* < 0.01).

**Table 3:** Patellar morphology after 25 mm from inferior pole in transverse cut

	Mean (mm)	SD	Max	Min
Width of patella (W)	43.25	4.76	53.21	25.15
Mid-anterior cortical thickness (MCOT)	4.04	1.01	6.01	1.11
Mid-cancellous thickness (MCAT)	14.26	2.12	18.24	8.68
Mid-cartilage thickness (MCRT)	1.08	0.46	2.53	0.47
Central ridge-anterior cortical thickness (CCOT)	4.77	0.98	6.68	1.98
Central ridge cancellous thickness (CCAT)	15.54	2.09	19.62	9.49
Central ridge cartilage thickness (CCRT)	1.55	0.65	3.9	0.71
Distance from mid-patella to central ridge (*N* = 46) (central ridge medial)	4.36	1.94	9.32	0.5
Distance from mid-patella to central ridge (*N* = 4) (central ridge lateral)	4.9	2.86	7.8	0.98

**Table 4:** Comparison between thickness and remaining cancellous bone after BPTB autograft at mid-patella and central-ridge location

	Mid-patella (mm)	Central ridge (mm)	Different (mm)	*P* value
Remaining cancellous bone (mean ± SD)	8.3 ± 2.04	10.31 ± 2.23	1.96	< 0.001
Anterior cortical bone thickness	4.04 ± 1.01	4.77 ± 0.98	0.73	< 0.001
Cancellous bone thickness	14.26 ± 2.12	15.54 ± 2.09	1.28	< 0.001
Cartilage	1.08 ± 0.46	1.55 ± 0.65	0.47	< 0.001

## Discussions

Patella fracture after BPTB graft harvesting is a rare complication, but it can lead to poor clinical outcome [6]. Patella fracture patterns have 2 common configurations. First, a longitudinal crack described as a “patella fissure” occurs after levering the graft from the patellar bed with an osteotome. The second type is a classic displacement of the transverse fracture that disrupts the extensor mechanism with high variability in shapes and fracture configurations. The relationship between a preexisting fissure fracture and transverse patella fracture has not been documented. It is likely that the displaced patella fractures can be caused by stress riser effect of the donor site defect [3]. Moholkar et al. advocated to harvest the BPTB at the sharp corner instead of at the round corner which has a higher chance of failure after the procedure [11]. The common BPTB harvesting technique uses patella bone graft that is 10 mm in width, 20–30 mm in length, and thickness usually less than 10 mm [8, 12, 13]. Some surgeons use the medial third BPTB harvesting technique based on Wiberg’s theory that the thickest central ridge was commonly located at the medial side of the patella. However, there were still reports of patella fractures after medial one-third BPTB harvesting [5]. We found out that the patella morphology has a high variability, and the best location for BPTB harvesting should be individualized.

According to Wiberg [9], there are 3 different types of patellar. Based on the axial radiographic view, we can see that type I has a concave, symmetrical, and of equal size facets. The second type of patellar medial facet is smaller than the lateral facet. The medial facet is flat or slightly convex and its lateral facet is concave. The third type of patellar has a convex medial facet that is markedly smaller than the concave lateral facet, and the angle between the medial and lateral facets is nearly 90°. There was another type of patella called “Jaegerhut” or Wiberg type IV that has no medial facet and median ridge [10]. We could not find any Jaegerhut in our study.

From our study, out of 50 cadavers, 14%, 68%, and 12% were of Wiberg’s types I, II, and III, respectively. Interestingly, we found 3 samples (6%) that had a larger medial facet compared to the lateral facet which did not match any of Wiberg’s classification.

Average patellar width, length, and thickness was 42.8 ± 5.25 mm, 32.75 ± 3.58 mm, and 21.63 ± 2.08 mm, respectively. Our finding was similar to the study by Huang et al. [14] which measured the patella morphology using CT imaging.

The average distance from the midline to the central patellar ridge was measured. The distance was 4.36 ± 1.94 mm in the medial central patellar ridge (92%) and 4.9 ± 2.86 mm in the lateral central patellar ridge (8%). This means that the thickest part of the patella (supposed to be the best area for BPTB graft harvesting) was located about 4.36 mm medially and 4.9 mm laterally, depending on the location of the central ridge. However, due to high variability between patients, therefore, the location for BPTB harvesting should be individualized. The location of the thickest bone should be assessed by axial patella radiographic view before harvesting the graft.

There were studies that correlated patellar morphology in patellar instability. The study by Servien et al. revealed that patellar Wiberg type III was more involved in patients with patellar dislocation [15]. The other study by Sandro compared patellar morphology in 22 trochlear dysplastic knees and 22 normal trochlear knees group by MRI imaging; the study results showed that trochlear dysplastic knee group had higher prevalence of a type II or III Wiberg’s classification (*p* = 0.02). Its overall size and the medial facet were smaller. However, there was no significance correlation between the type of trochlear dysplasia and patellar morphology [16]. In our study, most of the patellar are type II Wiberg. However, we did not correlate the morphology of the patella with the trochlear groove.

Harvesting BPTB from the central patellar ridge which had a thicker bone should decrease the risk of fracture. As to our knowledge, there were many studies which reported patellar fracture after BPTB harvesting in central third area ranging from 0.23 to 2.3% [2, 3, 6, 17], and there was only 1 study which reported patellar fracture after medial-third BPTB harvesting [5]. The fracture was reported in 4/478 patients (0.83%) in medial-third BPTB harvesting. The outcome reported by Marimuthu et al. of medial-third BPTB harvesting was comparable with the conventional middle-third harvesting. The advantage of medial-third BPTB harvesting discussed by the author included less risk of patellar tendon rupture/shortening, patellar fracture, and patellar mal-tracking [18]. The other advantage by the ultrasonographic study of medial-third BPTB harvesting was the patellar tendon that regained 95% of the cross-sectional area after 30 months [19]. Although the incidence of patellar fracture between central-third and medial-third BPTB harvesting were comparable, we believed that the reason behind this was due to the location of the thickest bone was not always in the medial third. Only 62% of the central patellar ridge shifted medially according to our study. However, the location of the central ridge alone might not be the only factor that causes patellar fracture; there were many risk factors that were associated with patellar fracture such as mechanism of injury, technique of harvesting, quality of bone, and many others.

**Fig. 5:**
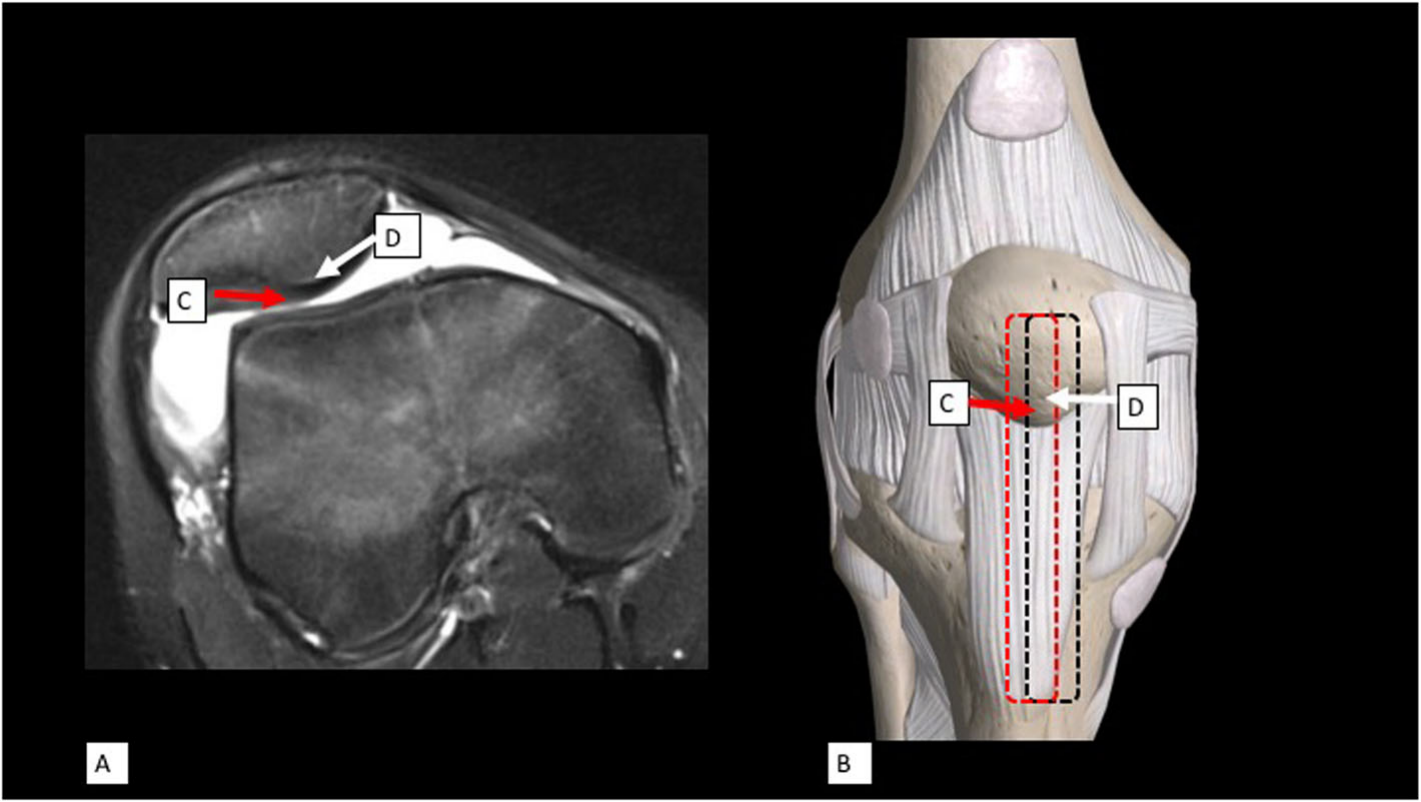
Method of pre-operative measurement of the harvesting area for bone-patella-tendon autograft. **a** Axial MRI of the right knee of a young male with anterior cruciate ligament (ACL) injury. Measurement of the distance from the mid-patella (**c**) to the central-ridge (**d**). **b** During the surgical procedure, the distance from the mid-patella to the central-ridge was measured (**c**, **d**). The new harvesting area (black dashed line) was shifted from the previous mid-patella area (red dashed line)

Our study confirmed that the remaining cancellous bone after harvesting 10-mm depth of BPTB at the central ridge was significantly thicker than in the mid-patella area (10 mm vs 8 mm). We recommend identifying the location of the central ridge from the axial patella imaging to locate the safest area to harvest BPTB graft. Intraoperatively, after exposure of the entire patellar tendon, the center of the harvesting area should be shifted medially or laterally according to the location of central patellar ridge which was seen from the axial MRI. For example, if the central ridge deviated 3 mm medially from the mid-patella, then, the center of the harvesting BPTB should be 3 mm medially from the middle of patella, patellar tendon, and tibial tuberosity from proximal to distal. If the decided width of the graft was 10 mm, marked the new center of harvesting area with a marker pen. From the new center of harvesting area, marked the incision line 5 mm medially and laterally and incised the BPTB as plan (Fig. 5).

Our study has some limitations. Since this is a cadaveric study, the sample size was small (*n* = 50). Aside from that, some data were difficult to interpret such as the level of deviation from the central ridge to the lateral side. Moreover, the transverse cut level of 25 mm from the inferior pole of the patella represented the thickness at the upper end of the harvesting area. We all know that the patellar thickness decreases throughout the distal pole resulting in fewer cancellous bone. Thus, the risk of fracture should be higher distally.

## Conclusion

The result of our study revealed that most of the central patellar ridge deviated medially, approximately 4 mm from the mid-patella. The thickest bone area was at the central patellar ridge. Harvesting BPTB graft at the central patella ridge area should have more remaining bone compared to the mid-patella area.

## Abbreviations

BPTB: Bone-patellar tendon-bone autograft
ACL: Anterior cruciate ligament
MRI: Magnetic resonance imaging
